# Editorial: Diet and digestive tract cancers: investigating the nutritional influences on gastrointestinal carcinogenesis

**DOI:** 10.3389/fnut.2025.1728806

**Published:** 2025-11-06

**Authors:** José María Huerta, Sandra Milena Colorado-Yohar, Diego Salmerón, Nelson Armando Agudelo

**Affiliations:** 1 CIBER Epidemiología y Salud Pública (CIBERESP), Madrid, Spain; 2 Department of Epidemiology, Murcia Regional Health Council, Murcia, Spain; 3 Murcia Biomedical Research Institute (IMIB), Murcia, Spain; 4 National Faculty of Public Health, University of Antioquia, Medellín, Colombia; 5 Department of Health and Social Sciences, University of Murcia, Murcia, Spain

**Keywords:** diet, colorectal cancer (CRC), gastrointestinal tumor, epidemiology, prevention, health inequalities, gastric cancer, lifestyles

## Introduction

In this Research Topic of “Frontiers in Nutrition”, we invited contributions that explore the complex relationships between diet, lifestyle, and gastrointestinal (GI) cancers. Our aim was to consolidate emerging evidence on established risk factors and to uncover novel dietary and behavioral determinants of carcinogenesis. Of particular importance are modifiable exposures—especially dietary and lifestyle habits—through which cancer risk can be reduced or the development of precancerous lesions prevented. The articles included in this issue primarily focus on preventable factors such as obesity and diet in relation to colorectal and gastric cancers, the most prevalent and concerning GI malignancies worldwide due to their rising incidence and high burden (1). The successful response to our call for articles underscores the enduring scientific and public health interest in the primary prevention of these cancers.

## Dietary risk factors and regional insights

A notable feature of this Research Topic is the strong representation of studies from Asian populations, where epidemiological data have often been less comprehensive. Several contributions identified local dietary practices that may increase risk, whether due to specific food items, their components, or cooking and consumption methods. For instance, Zheng et al. reported a positive association between fried soy consumption and colon polyp prevalence, while no associations were observed for boiled or marinated soy products in a large high-risk Chinese cohort. Prior studies in Asian populations have generally shown null or inverse associations between legume or soy food intake and colorectal cancer (CRC), but these analyses rarely considered cooking methods. The study highlights the intricate interplay between diet and CRC and reinforces the evidence suggesting potential adverse effects of fried foods on health.

Liao et al. conducted a meta-analysis of case–control studies assessing red and processed meat consumption and CRC risk among Asians. Contrary to the International Agency for Research on Cancer (IARC) classification of processed meats as carcinogenic to humans (Group 1) (2), their results showed a significant association for red meat only. The absence of a statistically significant relationship for processed meat likely reflects the limited number of studies (n=6) and the predominance of retrospective designs. Nonetheless, the findings reinforce the need for additional high-quality, population-specific evidence.

Iron metabolism has emerged as a key mechanistic pathway linking meat consumption and colorectal carcinogenesis. Yousefi et al. reviewed evidence suggesting that both excess and deficiency of iron may promote carcinogenesis through oxidative stress or altered immune responses. Therefore, prudent management of iron status should be considered in preventive and therapeutic strategies for CRC. In parallel, Shi et al., using data from the Prostate, Lung, Colorectal and Ovarian (PLCO) Cancer Screening Trial, examined the relationship between protein quality and colorectal adenoma risk. Their analysis showed that a higher Protein Diet Score—reflecting greater total protein intake and a higher plant-to-animal protein ratio—was inversely associated with adenoma incidence. This finding supports a dietary shift toward plant-based protein sources to maintain adequate protein intake while minimizing carcinogenic exposures.

## Global burden and dietary contributors

The Global Burden of Disease (GBD) framework has become an indispensable tool for quantifying the impact of diet and lifestyle on cancer outcomes. In this Research Topic, Yang et al. and Su et al. used GBD data to estimate the contribution of specific dietary risks—including low intake of milk, whole grains, calcium, and fiber—to the rising global burden of CRC. While age-standardized mortality rates and disability-adjusted life years (DALYs) associated with some dietary factors have declined, population growth and aging continue to drive an overall increase in absolute disease burden, particularly in low- and middle-income regions.

## Adiposity and colorectal cancer risk

Obesity remains a cornerstone modifiable risk factor in CRC prevention. Advances in anthropometric assessment now allow more precise characterization of adiposity beyond body mass index (BMI). Using data from over 100,000 participants in the US National Health and Nutrition Examination Survey (NHANES), Wang et al. demonstrated that relative fat mass (RFM)—an index derived from height and waist circumference—was strongly associated with CRC prevalence. The association was quasi-linear between 20 and 40 RFM units but diverged by sex at higher values, with stronger effects observed in younger adults (< 58 years). Similarly, Liu et al. found that the A Body Shape Index (ABSI) was a superior predictor of CRC prevalence compared with BMI or waist-to-height ratio, particularly among adults aged 40–60 years. These findings emphasize the need to consider alternative adiposity indices when evaluating metabolic risk in cancer epidemiology.

## Disparities, awareness, and prevention

The translation of evidence into preventive action remains challenging, particularly in regions with limited resources or awareness. He et al. analyzed GBD data to assess temporal trends in CRC burden by sociodemographic index (SDI) from 1994 to 2021. Their findings revealed persistent inequalities, with higher-income countries showing declining DALYs, while lower-SDI countries experienced increasing burdens due to restricted access to screening and early detection. In Saudi Arabia, Alkhaldy reported that nearly three-quarters of surveyed adults demonstrated poor CRC-related knowledge and unfavorable attitudes, particularly within vulnerable populations. Targeted educational initiatives and equitable access to screening programs are therefore essential to reduce disparities and improve outcomes.

## Gastric cancer and dietary determinants

Two additional studies examined nutritional factors in relation to gastric cancer (GC) across East Asia—where GC incidence remains among the world's highest. Using GBD data, Lui et al. documented declining age-specific GC mortality attributable to salt intake over the past three decades in China, Japan, and South Korea, yet salt consumption continues to be a leading contributor to GC burden, especially among older men. In the Korean context, Kim and Kim analyzed nearly 19,000 participants from the Korea National Health and Nutrition Examination Survey (KNHANES) and identified several dietary predictors of GC prevalence, including low intakes of protein and thiamine and the consumption of traditional foods such as “tteok” (rice cakes) and “soju” (a distilled rice beverage). These novel findings underscore the importance of culturally tailored dietary recommendations in GC prevention.

## Outlook

Despite the breadth and quality of research presented, the multifaceted relationship between diet and GI cancers cannot be exhaustively addressed within a single Research Topic. Most contributions focused on colorectal and gastric cancers, yet similar mechanisms may underlie malignancies of the liver, pancreas, and esophagus. Although current studies are primarily epidemiologic, future research integrating dietary pattern analyses with biomarker and molecular data will be crucial to clarify which dietary exposures are genuinely causal and to elucidate the biological pathways through which they may modulate gastrointestinal carcinogenesis. Continued epidemiologic and mechanistic research is essential to advance prevention strategies and mitigate the growing global burden of these diseases.
